# Supplementary material for: Spatiotemporal optical vortices with controllable radial and azimuthal quantum numbers
Source: Nat Commun. 2024 Jun 27;15:5435. doi: 10.1038/s41467-024-49819-4 (PMC11211508; doi:10.1038/s41467-024-49819-4)
Supplement: Supplementary file 1 — Supplementary information: Spatiotemporal optical vortices with controllable radial and azimuthal quantum numbers [file 41467_2024_49819_MOESM1_ESM.docx]

**Supplementary information: Spatiotemporal optical vortices with controllable radial and azimuthal quantum numbers**

Xin Liu^1,2,†^, Qian Cao^3,4,†^, Nianjia Zhang^3^, Andy Chong^5,6^, Yangjian Cai^1,2,*^, Qiwen Zhan^3,4,7,*^

^1^ Shandong Provincial Engineering and Technical Center of Light Manipulations and Shandong Provincial Key Laboratory of Optics and Photonic Device, School of Physics and Electronics, Shandong Normal University, Jinan 250014, China.

^2^ Collaborative Innovation Center of Light Manipulations and Applications, Shandong Normal University, Jinan, 250358, China.

^3^ School of Optical-Electrical and Computer Engineering, University of Shanghai for Science and Technology, Shanghai 200093, China.

^4^ Zhangjiang Laboratory, 100 Haike Road, Shanghai, 201204, China.

^5^ Department of Physics, Pusan National University, Busan, 46241, Republic of Korea.

^6^ Institute for Future Earth, Pusan National University, Busan, 46241, Republic of Korea

^7^ Westlake Institute for Optoelectronics, Fuyang, Hangzhou 311421, China.

^†^ These authors contributed equally to this work.

^*^ Corresponding authors: yangjiancai@sdnu.edu.cn; qwzhan@usst.edu.cn.

1. **Spatiotemporal Laguerre-Gaussian wavepackets**

In this section, we prove a 3D spatiotemporal Laguerre-Gaussian wavepacket is a stable solution of the scalar paraxial wave equation to an anomalous dispersive medium. Under the paraxial condition and slowly varying envelope approximation, the complex field of a scalar pulsed beam can be rewritten as

$u\left(x,y,z,t\right)=\Psi\left(x,y,z,t\right)e^{i\omega_{0}t-ik_{0}z},$ (1)

where $\Psi(x,y,z,t)$is the envelope of the pulsed beam, also called spatiotemporal wavepacket. The evolution dynamic of the spatiotemporal wavepackets in the dispersion medium is described by [1-3]

$\beta_{2}\frac{\partial^{2}\psi}{\partial\tau^{2}}-\frac{1}{k_{0}}\frac{\partial^{2}\psi}{\partial x^{2}}-\frac{1}{k_{0}}\frac{\partial^{2}\psi}{\partial y^{2}}-2i\frac{\partial\psi}{\partial z}=0,$ (2)

where $\tau=t-z/{v_{g}}$ is the local time frame and *v_g_* is the group velocity. $k_{0}={\omega_{0}}/c$ is the wavenumber. *β_2_* is the group velocity dispersion (GVD) coefficient of medium. For an anomalous dispersive medium with *β*_2_=-1/k_0_, Eqn. (2) can be reduced to a symmetric form in both space and time, which reads

$\frac{\partial^{2}\psi}{\partial\tau^{2}}+\frac{\partial^{2}\psi}{\partial x^{2}}+\frac{\partial^{2}\psi}{\partial y^{2}}+2i\chi\frac{\partial\psi}{\partial z}=0,$ (3)

where $\chi=1/{k_{0}=}{-\beta}_{2}$. Here, we assume the wavepacket has no coupling between *x-t* component and *y* component, the wavepacket can be separated into products of the two components, i.e. $\Psi\left( x,y,z,\tau\right)=\Psi_{ST}\left( \tau,x,z \right)\cdot\Psi_{Y}\left( y,z \right)$. As such, Eqn. (3) can be divided into two partial differential equations, are given by

$\frac{\partial^{2}\psi_{Y}}{\partial y^{2}}+2i\chi\frac{\partial\psi_{Y}}{\partial z}=0,$ (4)

$\frac{\partial^{2}\psi_{ST}}{\partial x^{2}}+\frac{\partial^{2}\psi_{ST}}{\partial\tau^{2}}+2i\chi\frac{\partial\psi_{ST}}{\partial z}=0$ (5)

A simplest solution of Eqn. (4) is a Gaussian function has a form of $\Psi_{Y}\left( y,z \right)=\left[ 1+iz/{z_{R}} \right]^{-1}\exp\left[ {iy^{2}z}/{w^{2}\left( z \right)z_{R}}-{y^{2}}/{w^{2}\left( z \right)} \right]$, where $w\left( z \right)=w_{0}\sqrt{1+\left( z/{z_{R}} \right)^{2}}$ and $z_{R}=\pi w_{0}^{2}/\lambda$ [4].To find a solution of Eqn. (5), we rewritten it in the cylindrical coordinates as

$\frac{1}{\rho^{2}}\frac{\partial^{2}\Psi_{ST}\left(\rho,\theta,z\right)}{\partial\theta^{2}}+\frac{1}{\rho}\cdot\frac{\partial}{\partial\rho}\left[\rho\cdot\frac{\partial\Psi_{ST}\left(\rho,\theta,z\right)}{\partial\rho}\right]+2i\chi\frac{\partial\Psi_{ST}\left(\rho,\theta,z\right)}{\partial z}=0,$ (6)

where $\rho=\sqrt{\tau^{2}+x^{2}}$ and $\theta=\tan^{-1}\left( x/\tau\right)$. We assume a trial solution of the form

$\Psi_{ST}=g\left[\frac{\rho}{w\left(z\right)}\right]\cdot h\left(\rho,\theta,z\right)=g\left[\frac{\rho}{w\left(z\right)}\right]\exp\left[-iP\left(z\right)+\frac{i}{2q\left(z\right)}\rho^{2}-il\theta\right].$ (7)

On substituting Eqn. (7) to Eqn. (6), we arrive at the following partial differential equation

$g''h+\left(\frac{2iw\rho}{q}+\frac{w}{\rho}-2i\rho w'\right)g'h+\left(2w^{2}P'+\frac{iw^{2}}{q}+\frac{w^{2}}{q^{2}}\rho^{2}\left(q'-1\right)-w^{2}\frac{l^{2}}{\rho^{2}}\right)gh=0,$ (8)

where the $g^{'}$ and $g''$ denotes the first and second derivatives with respect to corresponding variables. To make the equation to converge as $r\to\infty$, Eqn. (8) must satisfy $q^{'}=1$. Furthermore, we assume that

$\begin{matrix} q\left(z\right)=z+iz_{0}; \\ R\left(z\right)=z\left[1+\left(\frac{z_{0}}{z}\right)^{2}\right]; \\ w\left(z\right)=w_{0}\sqrt{1+\left(\frac{z}{z_{0}}\right)^{2}}, \end{matrix}$ (9)

where $z_{0}={w_{0}^{2}}/{2{\chi\lambda}_{0}}$. Substituting Eqn. (9) to Eqn. (8) and yields

$g''+\left[\frac{w\left(z\right)}{\rho}-\frac{4\rho}{w\left(z\right)}\right]g'+\left[2w^{2}P'+\frac{iw^{2}}{q}+\frac{l^{2}}{\rho^{2}}w^{2}\left(z\right)\right]g=0.$ (10)

The Eqn. (10) is very similar to the standard differential equation for the associated Laguerre polynomials $L_{p}^{l}(\nu)$, which has the form

 (11)

where $v={2\rho^{2}}/{w^{2}}$ and $p$ is a nonnegative real integer. Combined Eqn. (9) through Eqn. (11) with Eqn. (7), we can obtain

$\frac{\partial P\left(z\right)}{\partial z}=\frac{z_{0}}{z^{2}+z_{0}^{2}}\left(2p+\left|l\right|+1\right)-i\frac{z}{z^{2}+z_{0}^{2}},$ (12)

and

$\exp\left[-iP\left(z\right)\right]=\left[1+\left(\frac{z_{0}}{z}\right)^{2}\right]^{-\frac{1}{2}}\exp\left[-i\left(2p+\left|l\right|+1\right)\tan^{-1}\left(\frac{z}{z_{0}}\right)\right].$ (13)

A complete spatiotemporal solution, taken a spatiotemporal Laguerre-Gaussian (STLG) form in space-time domain, is thus given by

$\begin{matrix} \Psi\left(\rho,\theta,y,z\right)=\frac{1}{w\left(z\right)\left(1+iz/z_{0}\right)}\left[\frac{2p!}{\pi\left(\left|l\right|+p\right)!}\right]^{\frac{1}{2}}\exp\left[i\left(2p+\left|l\right|+1\right)\tan^{-1}\left(\frac{z}{z_{0}}\right)+i\frac{(\rho^{2}+y^{2})}{2\chi R\left(z\right)}\right] \\ \times\left[\frac{\sqrt{2}\rho}{w\left(z\right)}\right]^{\left|l\right|}\exp\left[-\frac{\rho^{2}+y^{2}}{w^{2}\left(z\right)}\right]L_{p}^{\left|l\right|}\left[\frac{2\rho^{2}}{w^{2}\left(z\right)}\right]\exp\left(-il\theta\right). \end{matrix}$ (14)

At *z*=0, the STLG wavepacket reads

$\Psi\left(\rho,\theta,y,0\right)\propto\left[\frac{\sqrt{2}\rho}{w_{0}}\right]^{\left|l\right|}\exp\left[-\frac{\rho^{2}+y^{2}}{w_{0}^{2}}\right]L_{p}^{\left|l\right|}\left[\frac{2\rho^{2}}{w_{0}^{2}}\right]\exp\left(-il\theta\right).$ (15)

To better visualize the STLG wavepacket, we plot its 3D iso-intensity profiles of Eqn. (15) for $p=1$, $l=+1$and$p=1, l=+2$ in space-time domain.


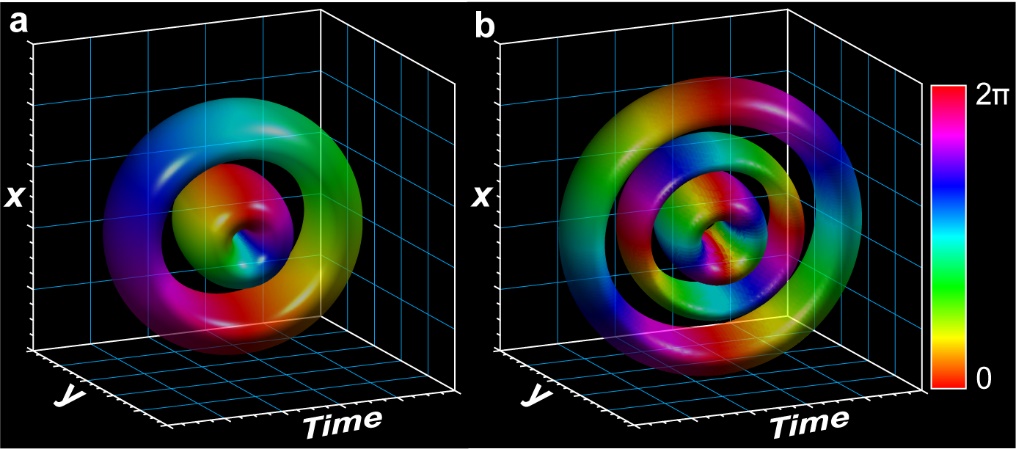


**Fig. S1.** Theoretical 3D iso-intensity profile of Eqn. (15) of **a,** $p=1, l=+1$ and **b,** $p=2, l=+2$ with 15% energy of the wavepacket in the isosurface. The iso-surface color denotes the phase distribution and the spatial and temporal coordinates are normalized to arbitrary units.

1. **Theory for synthesizing STLG wavepackets**

Under the paraxial approximation, the evolution properties of a scalar monochromatic beam propagating in free space can be studied with the help of the Fresnel integral [5]:

$u\left(x,y,L,\omega\right)=\frac{-i}{\lambda L}\int\int u_{0}\left(\xi,\eta,\omega\right)\exp\left\{\frac{ik_{0}}{2L}\left[\left(x-\xi\right)^{2}+\left(y-\eta\right)^{2}\right]\right\}d\xi d\eta,$ (16)

where$\xi$and$\eta$are the spatial coordinates at *L*=0 plane. For a pulsed beam $u\left( x,y,t,z \right)=\Psi\left( x,y,t,z \right)\exp\left( i\omega_{0}t-ik_{0}z \right)$ with a narrow bandwidth $\left| \Omega\right|=\left| \omega-\omega_{0} \right|\ll\omega_{0}$, its temporal envelope $\Psi\left( x,y,\tau,L \right)$ can be expressed as the Fourier transformation is [6]

$\Psi\left(x,y,t,L\right)=\frac{1}{2\pi}\int\Psi\left(x,y,\Omega,L\right)\exp\left(i\alpha\cdot\Omega^{2}\right)\exp\left(-i\Omega t\right)d\Omega,$ (17)

where $i\alpha\Omega^{2}$ is a group delay dispersion (GDD) phase induced by the dispersive elements. Thus, the spatiotemporal envelope of this 3D wavepacket at location *L* can be described by the combination of Eqn. (16) and (17), which reads

$\Psi\left(t,x,y,L\right)\simeq e^{ik_{0}\left[L+\left(x^{2}+y^{2}\right)/2L\right]}\int\int\int\Psi\left(\Omega,\xi,\eta,0\right)e^{ik_{0}\frac{\xi^{2}+\eta^{2}}{2L}-i\alpha\Omega^{2}}e^{-ik_{0}\frac{x\xi}{L}-i\Omega t}d\Omega d\xi d\eta.$ (18)

We now consider an input spatial-spectral LG seed beam at *L*=0 is given by

 (19)

where $r=\sqrt{\gamma^{2}\Omega^{2}+\xi^{2}}$ and $\varphi=\tan^{-1}\left( \xi/{\gamma\Omega} \right)$. $A_{0}$ is a normalized constant. Here, we are only interested in the wavepacket profile in the *x-t* plane and assume the wavepacket is distributed uniformly along the y-axis for simplicity. In the cylindrical coordinates, the Eqn. (18) can be rewritten as

 (20)

where$\alpha={\gamma^{2}k_{0}}/{2L}$, $\rho=\sqrt{\alpha^{2}x^{2}+\tau^{2}}$ and $\theta=\tan^{-1}\left( {\alpha x}/\tau\right)$. On substituting Eqn. (19) into (20) and using the following Bessel function and Laguerre integral formula [7]:

and

$$\int_{0}^{+\infty} x^{l+1}e^{-\alpha x^{2}}L_{p}^{l}\left(\beta x^{2}\right)J_{l}\left(xy\right)dx=2^{-l-1}\alpha^{-l-p-1}\left(\alpha-\beta\right)^{p}y^{l}e^{-\frac{y^{2}}{4\alpha}}L_{p}^{l}\left[\frac{\beta y^{2}}{4\alpha\left(\beta-\alpha\right)}\right].$$

We can arrive at the synthesized STLG wavepacket at location *L* with a spatiotemporal profile in space-time as

$\begin{matrix} \Psi_{\text{STLG}}(\rho,\theta)=2\pi i^{l}A_{0}L^{-1}\left(\sqrt{2}w_{1}^{-1}\right)^{l}\left(w_{2}/\sqrt{2}\right)^{l}2^{-l-1}\left(w_{1}^{-2}-i\alpha\right)^{-l-p-1}\left(-i\alpha-w_{1}^{-2}\right)^{p}e^{ik_{0}\left(L+x^{2}/2L\right)}e^{i\alpha w_{1}^{2}\rho^{2}/w_{2}^{2}} \\ \times\left(\frac{\sqrt{2}\rho}{w_{2}}\right)^{l}\exp\left(-\frac{\rho^{2}}{w_{2}^{2}}\right)L_{p}^{\left|l\right|}\left(\frac{2\rho^{2}}{w_{2}^{2}}\right)\exp\left(-il\theta\right), \end{matrix}$ (21)

where $\rho=\sqrt{\tau^{2}+\alpha^{2}x^{2}}$, $\theta=\tan^{-1}\left( {\alpha x}/\tau\right)$ and $w_{2}=2\sqrt{w_{1}^{-2}+\alpha^{2}w_{1}^{2}}$.

1. **Transverse OAM density analysis of STLG wavepacket**

In this section, we analyze the transverse angular momentum density of an STLG wavepacket. According to references [8-10], the angular momentum density is defined as $\mathbf{L}=\varepsilon\vec{\rho}\times(\vec{E}\times\vec{B})$. For a linearly polarized light $u$, the time-averaged linear momentum density is [8]

$\vec{g}=i\omega\frac{\varepsilon}{2}\left(u^{*}\nabla u-u\nabla u^{*}\right)+\omega k\varepsilon\left|u\right|^{2}\hat{z}$ (22)

where $\nabla=\left( \frac{\partial}{\partial\rho}-\frac{1}{\rho\tan\theta}\frac{\partial}{\partial\theta} \right)\hat{\rho}-\left( \frac{\partial}{\partial\rho}\tan\theta-\frac{1}{\rho}\frac{\partial}{\partial\theta} \right)\hat{\theta}+\frac{\partial}{\partial y}\hat{y}$ in the cylindrical coordinates; $\hat{\rho}, \hat{\theta}$ and $\hat{y}$ denotes unit vector along radial, azimuthal and *y* direction, respectively. The STLG wavepacket with a quantum number $p$ can be decomposed into a linear superposition of *p* components according to the following formula [7]

$L_{p}^{\left|l\right|}\left(v\right)=\sum_{m=0}^{p} \left(-1\right)^{m}\left(\begin{matrix} p+\left|l\right| \\ p-m \end{matrix}\right)\frac{v^{m}}{m!}.$ (23)

As such, we consider an STLG wavepacket with $p=1$ as an example, it can be rewritten as $\Psi_{1}^{l}\left( \rho,\theta,y \right)=u=u_{1}+u_{2}$ with

$u_{1}=\left(\sqrt{2}\right)^{l}\left(\left|l\right|+1\right)\frac{\rho^{l}}{w_{0}^{l}}\exp\left(-\frac{\rho^{2}}{w_{0}^{2}}\right)\exp\left(-\frac{y^{2}}{w_{0}^{2}}\right)\exp\left(-il\theta\right)$ (24)

$u_{2}=\exp\left(i\pi\right)\left(\sqrt{2}\right)^{l}\frac{2\rho^{l+2}}{w_{0}^{l+2}}\exp\left(-\frac{\rho^{2}}{w_{0}^{2}}\right)\exp\left(-\frac{y^{2}}{w_{0}^{2}}\right)\exp\left(-il\theta\right)$ (25)

Fig. S2a and b show their 3D iso-intensity surfaces and phase distributions for better intuitive. As shown, we call the ST wavepacket of Eqn. (24) as an inner ST wavepacket and call the ST wavepacket of Eqn. (25) as an outer ST wavepacket for convenience. Eqn. (24) and Eqn. (25) has the following relationship

$u_{2}=\frac{-2}{\left(\left|l\right|+1\right)w_{0}^{2}}\rho^{2}u_{1}.$ (26)

We can rewrite $u^{*}\nabla u-u\nabla u^{*}$ as

$u^{*}\nabla u-u\nabla u^{*}=u_{1}^{*}\nabla u_{1}-u_{1}\nabla u_{1}^{*}+u_{2}^{*}\nabla u_{2}-u_{2}\nabla u_{2}^{*}+\left(u_{1}^{*}\nabla u_{2}-u_{1}\nabla u_{2}^{*}+u_{2}^{*}\nabla u_{1}-u_{2}\nabla u_{1}^{*}\right),$ (27)

Substituting from Eqn. (24) to Eqn. (26) into Eqn. (27), we have

$u_{1}^{*}\nabla u_{2}-u_{1}\nabla u_{2}^{*}=\left[i\frac{4le^{i\pi}}{\left(\left|l\right|+1\right)w_{0}^{2}\tan\theta}\rho\left|u_{1}\right|^{2}\right]\hat{\rho}-\left[i\frac{4le^{i\pi}}{\left(\left|l\right|+1\right)w_{0}^{2}}\rho\left|u_{1}\right|^{2}\right]\hat{\theta},$ (28)

and

$u_{2}^{*}\nabla u_{1}-u_{2}\nabla u_{1}^{*}=\left[i\frac{4le^{i\pi}}{\left(\left|l\right|+1\right)w_{0}^{2}\tan\theta}\rho\left|u_{1}\right|^{2}\right]\hat{\rho}-\left[i\frac{4le^{i\pi}}{\left(\left|l\right|+1\right)w_{0}^{2}}\rho\left|u_{1}\right|^{2}\right]\hat{\theta},$ (29)

and

$u_{1}^{*}\nabla u_{1}-u_{1}\nabla u_{1}^{*}=\left[i\frac{2l}{\rho\tan\theta}\left|u_{1}\right|^{2}\right]\hat{\rho}-\left[i\frac{2l}{\rho}\left|u_{1}\right|^{2}\right]\hat{\theta},$ (30)

and

$u_{2}^{*}\nabla u_{2}-u_{2}\nabla u_{2}^{*}=\left[i\frac{8l}{\left(\left|l\right|+1\right)^{2}w_{0}^{4}\tan\theta}\rho^{3}\left|u_{1}\right|^{2}\right]\hat{\rho}-\left[i\frac{8l}{\left(\left|l\right|+1\right)^{2}w_{0}^{4}}\rho^{3}\left|u_{1}\right|^{2}\right]\hat{\theta}.$ (31)

We introduce a composited linear momentum density, takes the form

$\vec{g}=\vec{g}_{in}+\vec{g}_{out}+\vec{g}_{coup},$ (32)

with

$$\vec{g}_{in}=i\omega\frac{\varepsilon}{2}\left(u_{1}^{*}\nabla u_{1}-u_{1}\nabla u_{1}^{*}\right)+\omega k\varepsilon_{0}\left|u_{1}\right|^{2}\hat{z};$$

$$\vec{g}_{out}=i\omega\frac{\varepsilon}{2}\left(u_{2}^{*}\nabla u_{2}-u_{2}\nabla u_{2}^{*}\right)+\omega k\varepsilon_{0}\left|u_{2}\right|^{2}\hat{z};$$

$$\vec{g}_{coup}=i\omega\varepsilon\left(u_{1}^{*}\nabla u_{2}-u_{1}\nabla u_{2}^{*}\right)+2\omega k\varepsilon_{0}u_{2}u_{1}^{*}\hat{z},$$

where $\hat{z}=\hat{\rho}\cos\theta-\hat{\theta}\sin\theta$. Combined Eqn. (28) through Eqn. (31) and Eqn. (26), the corresponding linear momentum expressions are calculated as

$$\begin{matrix} \vec{g}_{in}=i\omega\frac{\varepsilon}{2}\left\{\left[i\frac{2l}{\rho\tan\theta}\left|u_{1}\right|^{2}\right]\hat{\rho}-\left[i\frac{2l}{\rho}\left|u_{1}\right|^{2}\right]\hat{\theta}\right\}+\omega k\varepsilon_{0}\left|u_{1}\right|^{2}\left(\hat{\rho}\cos\theta-\hat{\theta}\sin\theta\right), \\ \vec{g}_{out}=i\omega\frac{\varepsilon}{2}\left\{\left[i\frac{8l}{\left(\left|l\right|+1\right)^{2}w_{0}^{4}\tan\theta}\rho^{3}\left|u_{1}\right|^{2}\right]\hat{\rho}-\left[i\frac{8l}{\left(\left|l\right|+1\right)^{2}w_{0}^{4}}\rho^{3}\left|u_{1}\right|^{2}\right]\hat{\theta}\right\}+\frac{4\omega k\varepsilon_{0}}{\left(\left|l\right|+1\right)^{2}w_{0}^{4}}\rho^{4}\left|u_{1}\right|^{2}\left(\hat{\rho}\cos\theta-\hat{\theta}\sin\theta\right), \\ \vec{g}_{coup}=i\omega\varepsilon\left\{\left[i\frac{4le^{i\pi}}{\left(\left|l\right|+1\right)w_{0}^{2}\tan\theta}\rho\left|u_{1}\right|^{2}\right]\hat{\rho}-\left[i\frac{4le^{i\pi}}{\left(\left|l\right|+1\right)w_{0}^{2}}\rho\left|u_{1}\right|^{2}\right]\hat{\theta}\right\}+\frac{4\omega k\varepsilon_{0}\rho^{2}e^{i\pi}}{\left(\left|l\right|+1\right)w_{0}^{2}}\left|u_{1}\right|^{2}\left(\hat{\rho}\cos\theta-\hat{\theta}\sin\theta\right), \end{matrix}$$

The OAM density definition is $\mathbf{L}=\vec{s}\times\vec{g}$ where $\vec{s}=\rho\hat{\rho}+y\hat{y}$ [10]. As such, we can calculate each transverse OAM paralleled *y*-axis are

$\mathbf{L}_{in}=\omega\varepsilon\left|u_{1}\right|^{2}\left[l-k\rho\sin\theta\right]\hat{y}$ (33)

$\mathbf{L}_{out}=\frac{4\varepsilon\omega\rho^{4}\left|u_{1}\right|^{2}}{\left(\left|l\right|+1\right)^{2}w_{0}^{4}}\left[l-k\rho\sin\theta\right]\hat{y}$ (34)

$\mathbf{L}_{coup}=-\frac{4\omega\varepsilon\rho^{2}\left|u_{1}\right|^{2}}{\left(\left|l\right|+1\right)w_{0}^{2}}\left[l-k\rho\sin\theta\right]\hat{y}$ (35)

with the following relations for their magnitude

$\frac{\left|\mathbf{L}_{out}\right|}{\left|\mathbf{L}_{in}\right|}=\frac{4\rho^{4}}{\left(\left|l\right|+1\right)^{2}w_{0}^{4}}$ (36)

$\left|\mathbf{L}_{coup}\right|^{2}=4\left|\mathbf{L}_{in}\right|\cdot\left|\mathbf{L}_{out}\right|$ (37)

From Eqns. (33)-(37), we find that the total transverse OAM density is $\mathbf{L}_{OAM}=\mathbf{L}_{+y}+\mathbf{L}_{-y}$ where $\mathbf{L}_{+y}=\mathbf{L}_{in}+\mathbf{L}_{out}$ and $\mathbf{L}_{\boldsymbol{-}y}=\mathbf{L}_{coup}$.

Now, we try to gain some valuable physical insights by examining the formulas (33)-(37). (**i**) The total OAM along the *y*-axis consists of three terms, resulting from the inner ST wavepacket, the outer ST wavepacket and their coupled joint, respectively. In Eqns. (33)-(35), $l$ is for the intrinsic transverse OAM of the STLG wavepacket and its integration to $\theta$ is nonzero. (**ii**) Both the inner and the outer ST wavepackets carry individual transverse OAM with the same direction (*+y* for $l$>0), their magnitude ratio is described by Eqn. (36). However, existing a direction-*opposite* transverse OAM with a magnitude of Eqn. (37) in STLG wavepacket which is caused by the coupling of the inner and outer ST-wavepackets. The minus (*opposite-*direction) comes from the phase difference $\pi$ between the rings. (**iii**), the two ST wavepackets couple to each other, leading to a double vortex ring structure, as shown in Fig. S2c. Consequently, such STLG has a space-time cylinder-shaped phase edge dislocation with a zero-intensity and undefined phase at position $\rho=w_{0}\sqrt{\left( \left| l \right|+1 \right)/2}$ where the *opposite* transverse OAM reaches the maximum and the total transverse OAM is zero, as shown in Fig. S2d.


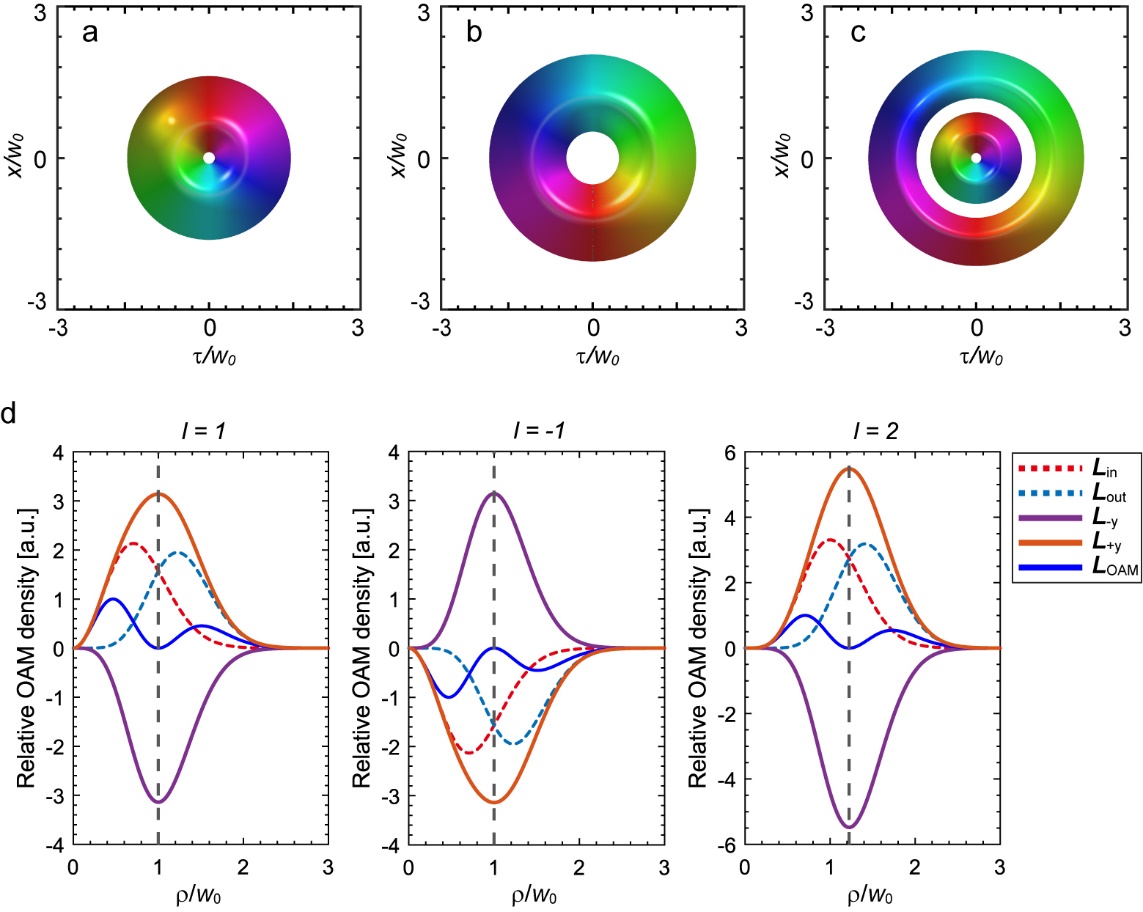


**Fig. S2.** **An STLG wavepacket of *p*=1 is decomposed into an inner ST wavepacket of Eqn. (24) and an outer ST wavepacket of Eqn. (25).** **a** and **b,** The 3D iso-intensity surface of (**a**) the inner ST wavepacket and (**b**) the outer wavepacket. **c,** The 3D iso-intensity surface of the STLG wavepacket results from a linear superposition of these two ST wavepackets (**a** and **b**). The isosurface color denotes the phase distribution. The isovalue is set to 0.15. **d,** The relative transverse OAM density distributions of an STLG wavepacket of $p=1$ with different azimuthal indices $l$. The vertical black dot line denotes the position $\rho=w_{0}\sqrt{\left( \left| l \right|+1 \right)/2}$.

1. **Numerical Propagation of an STLG wavepacket in** **the anomalous dispersive medium**

The spatiotemporal evolution dynamics of a wavepacket propagates in a dispersive medium can be numerically studied by the angular spectrum propagation theorem by means of the fast Fourier transformation method, given by [6,11]

$\Psi\left(\tau,x,y,z\right)=\frac{e^{ik_{0}z}}{8\pi^{3}}\iint\int\tilde{\Psi}_{\text{STLG}}\left(\Omega,k_{x},k_{y}\right)H\left(\Omega,k_{x},k_{y}\right)\exp\left(-ik_{x}x-ik_{y}y-i\Omega\tau\right)d\Omega dk_{x}dk_{y}$ (38)

where $H\left( \Omega,k_{x},k_{y} \right)=exp\left[ -i{\left( k_{x}^{2}+k_{y}^{2} \right)z}/{2k_{0}} \right]\exp\left( {i\beta_{2}\Omega^{2}z}/2 \right)$ is the transfer function of the dispersive medium and $\tilde{\Psi}_{STLG}\left( \Omega,k_{x},k_{y} \right)$ is the two-dimensional Fourier transform of $\Psi_{\mathrm{STLG}}\left( t,x,y \right)e^{{-ik_{0}\left( x^{2}+y^{2} \right)}/{2L}}$, where the later exponential term is to eliminate the residual spatial diffraction during the synthesis process. $k_{x}$ and $k_{y}$ are spatial frequencies in *x* and *y* directions. *β_2_* is the group velocity dispersion coefficient of the dispersive medium. For balancing the spatial diffraction and temporal dispersion, a medium with anomalous GVD ${\beta_{2}=-1}/{k_{0}}$ is chosen in the simulation. Fig. S3 shows the numerical simulation results of the generated STLG wavepacket propagates in an anomalous dispersive medium of $\beta_{2}=-24\mathrm{ps}^{2}/mm$ at different propagation distances. As shown, the STLG wavepacket shows a self-similar property and maintains its spatiotemporal topology profile after propagating a long distance.


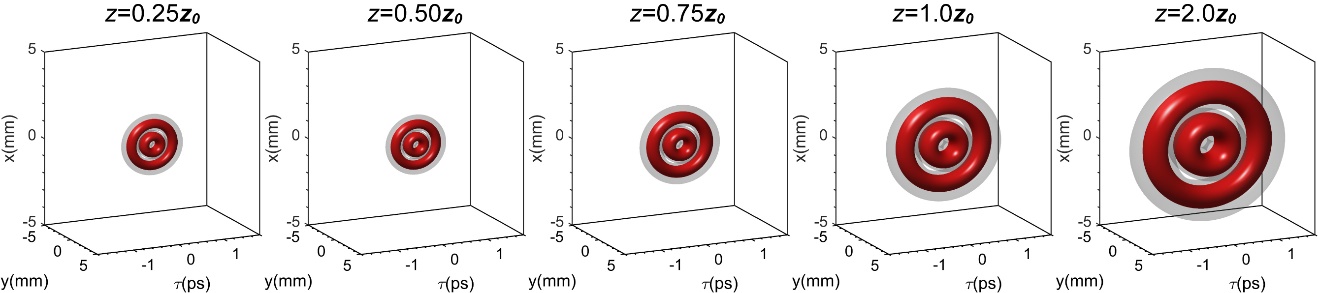


**Fig. S3. Numerical simulation for spatiotemporal evolution dynamic of the generated STLG wavepacket of** $\boldsymbol{p=1}$ **and** $\boldsymbol{l=+2}$ **in an anomalous dispersive medium of** $\boldsymbol{\beta}_{\boldsymbol{2}}\boldsymbol{=-24}\mathbf{ps}^{\mathbf{2}}\mathbf{/mm}$**. The** $\boldsymbol{z}_{\boldsymbol{0}}\boldsymbol{=}{\boldsymbol{\pi}\boldsymbol{w}_{\boldsymbol{2}}^{\boldsymbol{2}}}/\boldsymbol{\lambda}\boldsymbol{\approx1.95}$**m in this case.**

1. **Mode conversion of STLG wavepackets to STHG wavepackets**

The spatiotemporal astigmatism can be introduced by applying a spatial-spectral cross phase $\exp(i\mu\xi\Omega)$ [12,13] on the frequency spectrum of the STLG wavepacket, that is Eqn. (2) of the main text. In the case of ${\mu=2\gamma}/{w_{1}^{2}}$ and $\alpha\to0$, Eqn. (21) in the cartesian coordinates reduces to

 (39)

where $C_{0}={{w_{1}}^{2}}/{2^{p+2}\pi\gamma p!\sqrt{2}i^{p+l}}$. $H_{p}\left( \cdot\right)$ denotes the Hermite polynomials of *p*-order.

1. **Complex-amplitude modulation in the space-time domain via spatial filtering**

**
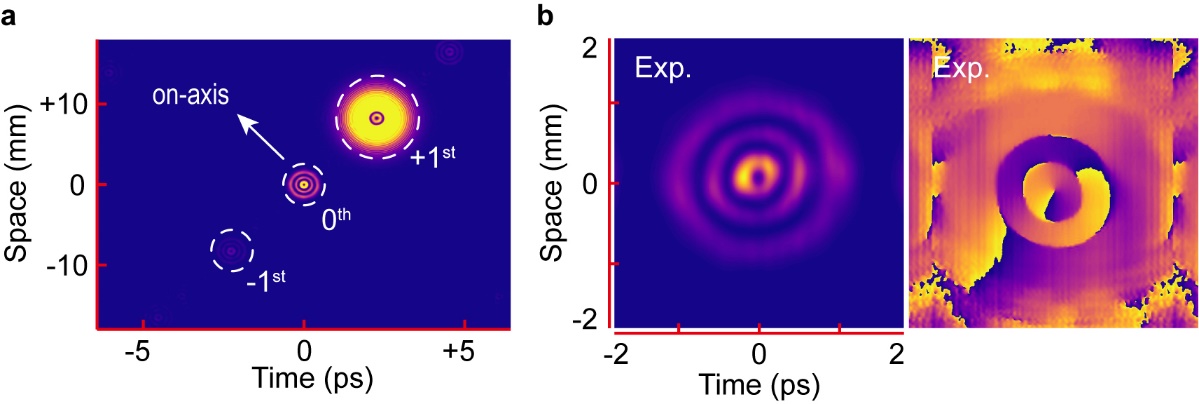
**

**Fig. S4. a,** After the grating, the light field reflected from the hologram is diffracted across both spatial and temporal domains. The target ST-wavepacket is situated within the on-axial zeroth diffraction order. **b,** Intensity (left) and phase (right) distributions of experimentally synthesized STLG wavepacket filtered by an iris, take *p*=2 and *l*=1 as an example.

1. **Characterization of laser source and probe pulse**


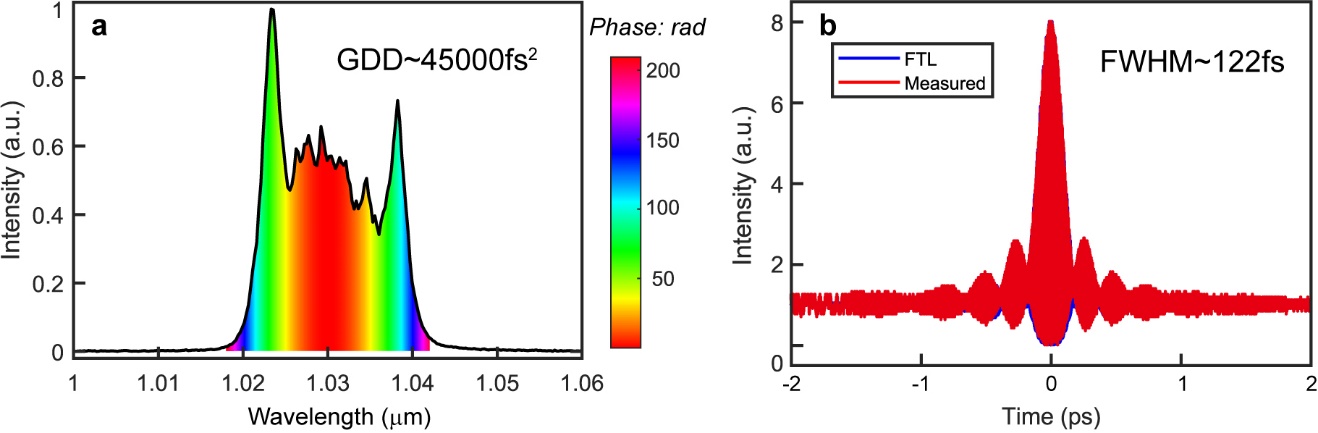


**Fig. S5.** **a,** The power spectrum of our lab-built mode-locked Yb: fiber femtosecond laser which has a ~20nm spectral width at a center wavelength of 1030nm and emits a chirped pulse with a positive GDD of ~45000fs^2^. **b,** The second-order autocorrelation interference fringe curves, the blue curve is the 0 GDD spectrum Fourier-transform limited (FTL) pulse, the red curve is the de-chirped pulse (measured FWHM is ~122fs) after a pulse compressor system, it closes to the FTL pulse.

1. **Characterization of STLG wavepackets using 3D diagnostic measurement technique**

The de-chirped probe pulse combines with the object pulse at a little angle (*ε*~0.6°) leading to a tilted interference wavefront (in the *y-t* plane) with respect to the object pulse, as shown in Fig. S6a. The probe pulse is considerably shorter and interferes with each temporal slice of the STLG wavepacket by scanning the probe pulse along the time axis with the help of a motorized translation stage, generating a series of time-dependent spatial interference fringe patterns, as shown in Fig. S6b-h. A CMOS camera records such interference fringe patterns those are given by [14]

$I\left(\tau,x,y\right)=I_{o}\left(x,y\right)+I_{p}\left(x,y\right)+2\sqrt{\Delta t_{p}\left|u_{o}\left(\tau,x,y\right)\right|^{2}I_{p}\left(x,y\right)}\cos\left[\delta\left(\tau,x,y\right)+k_{0}y\sin\varepsilon\right],$ (40)

where $I_{o}\left( x,y \right)$ and $I_{p}\left( x,y \right)$ are the time-integrated intensity distribution of the object and probe pulse beam, respectively. $\tau$ is the time delay of the probe pulse with respect to the object pulse. $\varepsilon$ is the tilted angle between the object and probe pulse beam. $\Delta t_{p}$ is the duration of the probe pulse. $\delta\left( \tau,x,y \right)$ is the transverse space-variant phase difference between the object pulse and the probe pulse at the temporal slice $\tau$. $u_{o}(\tau,x,y)$ is the complex filed information of the wavepacket at each temporal slice, which reads

$u_{o}\left(\tau,x,y\right)=A_{c}\left(\tau,x,y\right)/\sqrt{I_{p}\left(x,y\right)},$ (41)

where $A_{c}(\tau,x,y)$ is the coupling term in Eqn. (40) which can be obtained by low-pass filtering out the positive first order of $I(\tau,x,y)-I_{o}(x,y)-I_{p}(x,y)$ in the spatial frequency domain. Finally, we can reconstruct the three-dimensional spatiotemporal wavepacket by stitching all temporal slices of $\tau_{n}$

$\Psi_{3D}\left(\tau,x,y\right)=\sum_{n=1}^{N} u\left(\tau_{n},x,y\right),$ (42)

where *N* is the number of all temporal slices and is set to 100 in our experiment. The interval between adjacent temporal slices is ~33fs.

Fig. S6b-h shows some representative raw interference fringe patterns, take the STLG of$p=1$ and $l=+1$ as an example. Each pattern shows vertical fringes perpendicular to the *y-*axis whose visibility reflects the intensity distribution and the fringe shift reflects the phase information, respectively. When the probe pulse was scanned towards the STLG center from -264fs to 0fs, the fringes are first bended and then fully dislocated. The interference fringe exhibits three discontinuous phase jumps and intensity dimmish around the centered phase singularity, as shown in Fig. S6e, due to the double-ring structure of STLG wavepacket for $p=1$. When the probe pulse was scanned away from 0fs to 264fs, the fringes become vertical again. Such fringes evolution is a feature of an STLG wavepacket of $p=1$ and$l=+1$. It is worth mentioning that the fork-shaped (not uniform) dislocations of the fringes along the y-axis, observed at a fixed temporal slice, is attributed to the tilted angle (*ε*~0.6°) when interfering. Supplementary Movie 1 presents the three-dimensional reconstruction process of an STLG wavepacket.

**
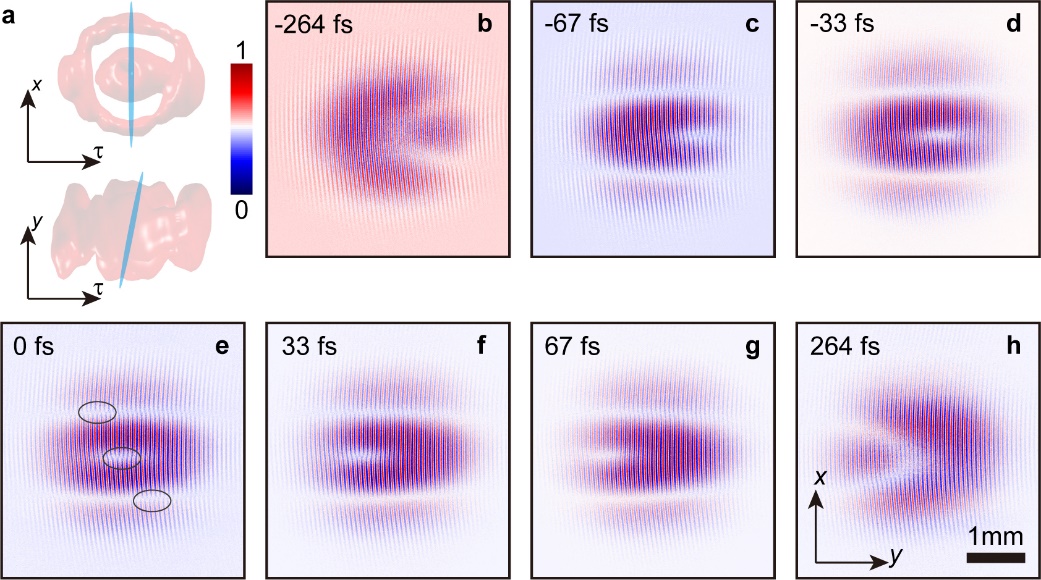
**

**Fig. S6. Measured interference fringes for characterization of the synthesized STLG wavepacket by scanning probe pulse. a,** Interference scheme between probe pulse and object wavepacket in different views. **b-h,** Representative raw interference fringe patterns of an STLG wavepacket of $p=1$ and $l=+1$ at various time delay.

1. **Modal analysis of the synthesized STLG wavepackets**

To estimate modal purity of the synthesized STLG wavepackets, we can analyze the modal weight by decomposing it into a set of LG modes, which reads [15,16]

$u\left(\tau,x\right)=\sum_{p=0}^{\infty} \sum_{l=-\infty}^{\infty} c_{p}^{l}\psi_{p}^{l}\left(\tau,x\right),$ (43)

where $c_{p}^{l}$ is a complex value and stands for modal weight on radial index $p$ and azimuthal index $l$. $\psi(\tau,x)$ is a set of complete orthonormal basis (${LG}_{p}^{l}$). The modal weight coefficient $\left| c_{p}^{l} \right|^{2}$ can be calculated from

 (44)

where * denotes the complex conjugate. Fig. S7 shows the normalized weight coefficients of the synthesized STLG wavepackets with different $p$ and $l$ in our experiment.


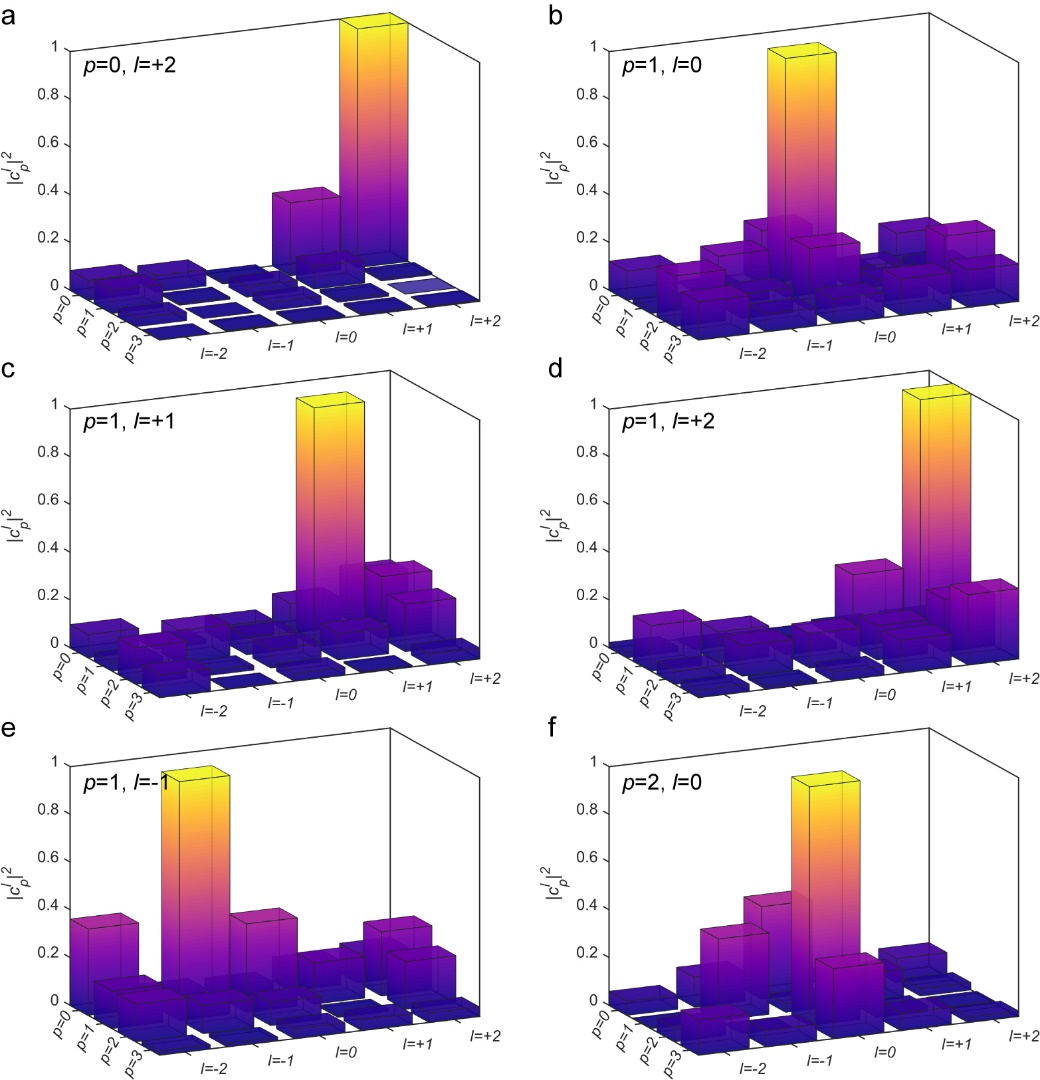


**Fig. S7. Modal weight analysis for the synthesized STLG wavepackets with different *p* and *l* for supporting the results of Fig. 4 in the main text.**

1. **Spatiotemporal intensity and phase distributions of the STLG wavepackets**


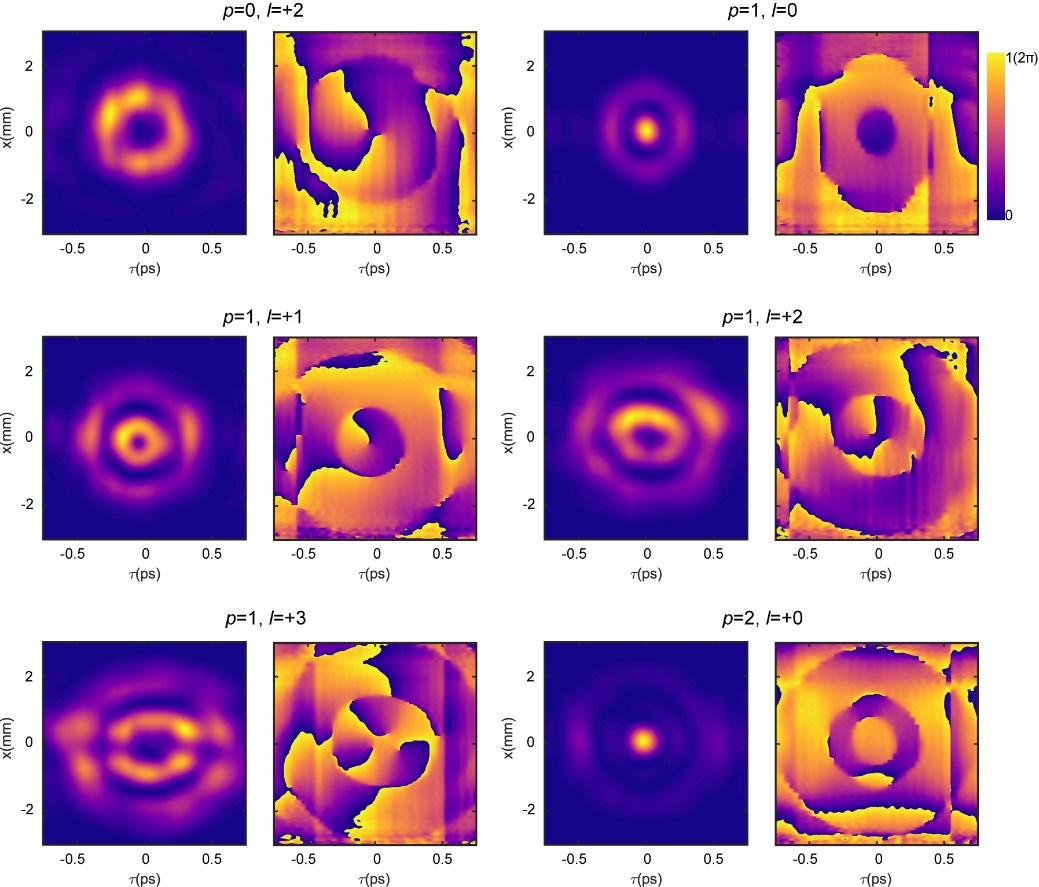


**Fig. S8. The corresponding accumulated intensity** $\int{\boldsymbol{|}\boldsymbol{\Psi}_{\boldsymbol{STLG}}\boldsymbol{(\tau,x,y)|}}^{\boldsymbol{2}}\boldsymbol{dy}$ **(left) and sliced phase at y=0 (right) distributions of the STLG wavepackets with different *p* and *l* for supporting the results of Fig. 4 in the main text.**

1. **Synthesizing STLG wavepackets with large *p* and *l***

Since the STLG wavepacket, presented in our work, is synthesized in the far-field (free propagation around L=1.2m after the pulse shaper in the experimental setup), thus for a given bandwidth, the STLG wavepackets with large *p* and *l* will undergo rapid spatial spreading after the pulse shaper, requiring a larger aperture camera to record. To address this issue, we can manipulate the focus phases (spatial and temporal focus) to break such limitations. In Fig. S9, we show some experimental examples of STLG wavepackets with large *p* and *l* to alleviate spatial broaden.


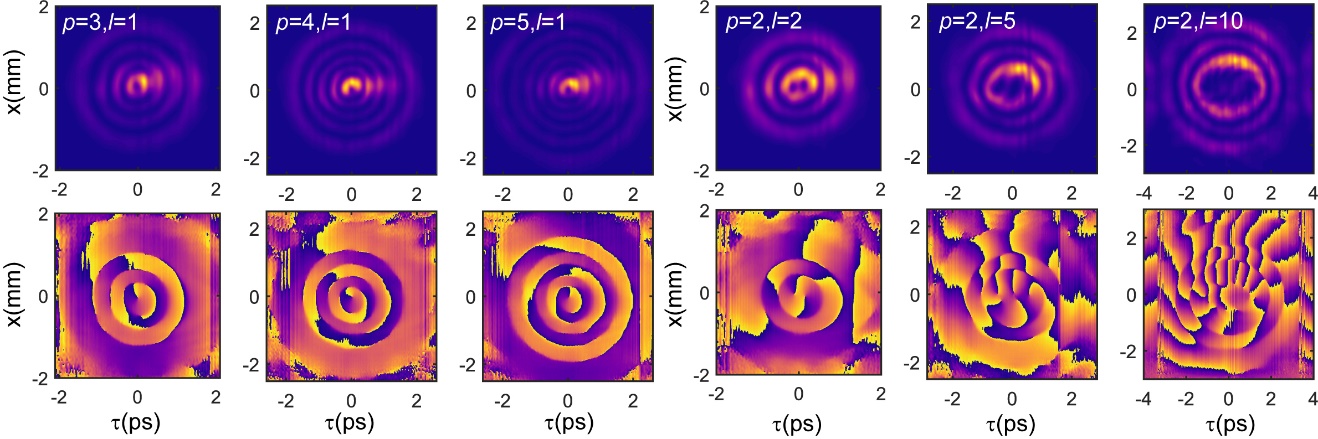


**Fig. S9. Experimental examples for the generation of STLG wavepackets with large *p* and *l* via manipulating the diffraction and dispersion phases on the SLM (by adding focus phases to alleviate ST broaden in the experiment, carried GDD~23000fs^2^). Top: Intensities; Bottom: Phases.**

1. **Spatiotemporal intensity and phase distributions of the converted STHG wavepackets**


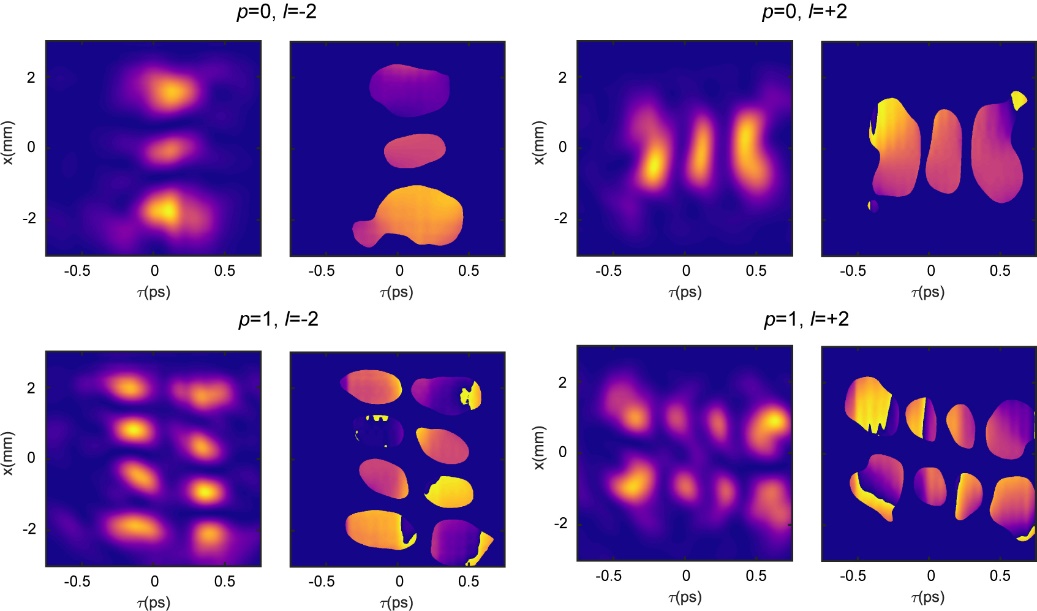


**Fig. S10. The corresponding accumulated intensity** $\int{\boldsymbol{|}\boldsymbol{\Psi}_{\boldsymbol{STHG}}\boldsymbol{(\tau,x,y)|}}^{\boldsymbol{2}}\boldsymbol{dy}$ **(left) and sliced phase (y=0) distributions of the converted STHG wavepackets for different *p* and *l* for supporting the results of Fig. 5 in the main text.**

1. **Mode conversion of STLG to STHG with different spatiotemporal astigmatism strength**

In Fig. S11, we display the experimental process of mode conversion of STLG wavepacket of *p*=1 and *l*=+2 to STHG wavepacket with different spatiotemporal astigmatism strength.


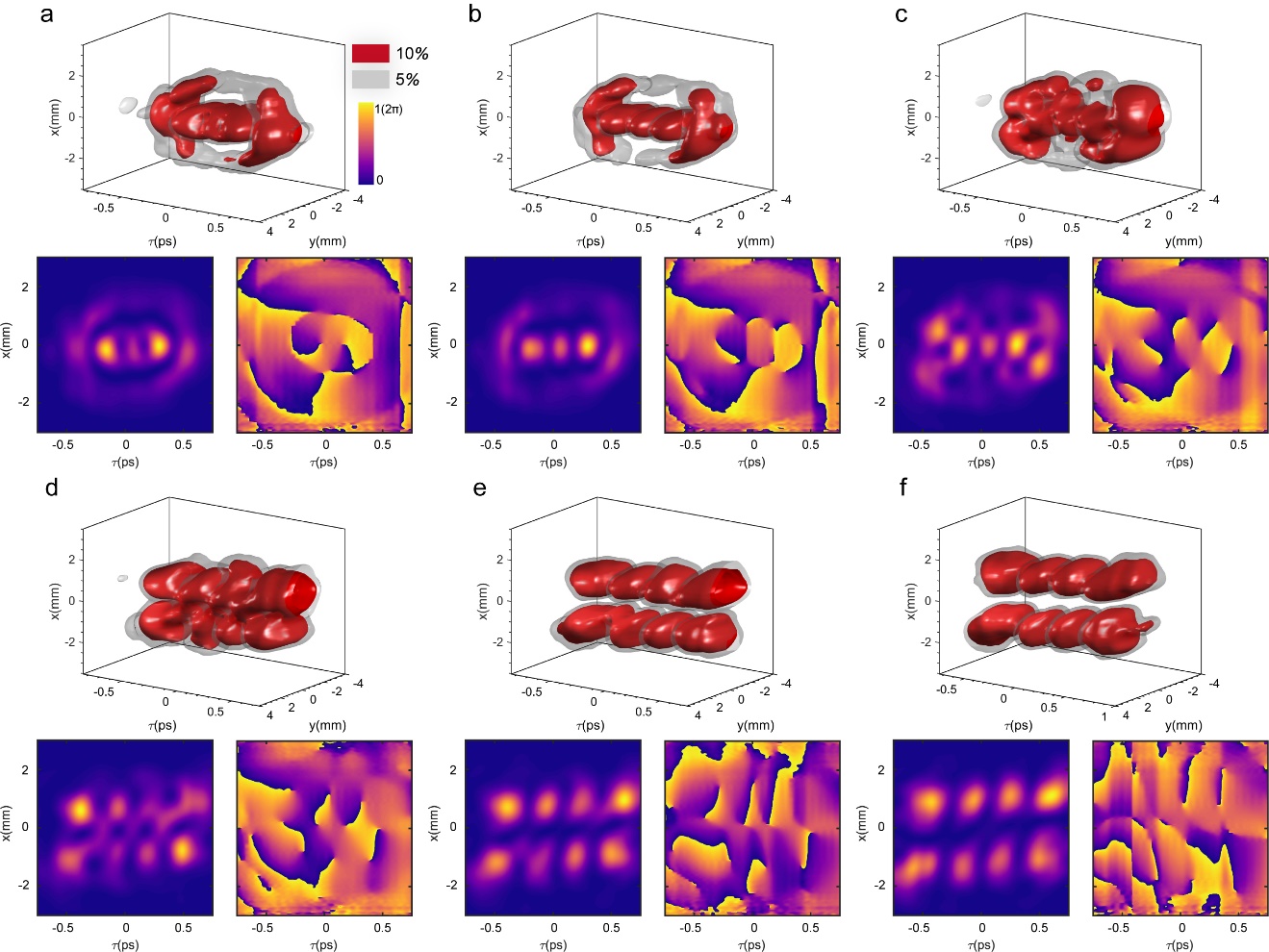


**Fig. S11. Mode conversion of STLG to STHG of *p*=1 and *l*=+2 with different spatiotemporal astigmatism strength. a,** $\mu\boldsymbol{=}2fs/(rad\cdot mm)$; b, $\mu\boldsymbol{=}4fs/(rad\cdot mm)$; c, $\mu\boldsymbol{=}6fs/(rad\cdot mm)$; d, $\mu\boldsymbol{=}8fs/(rad\cdot mm)$; e, $\mu\boldsymbol{=}10fs/(rad\cdot mm)$ and f, $\mu\boldsymbol{=}12fs/(rad\cdot mm)$.

**Supplementary References**

1. Borovkova, O. V., Kartashov, Y. V., Lobanov, V. E., Vysloukh, V. A. & Torner, L. General quasi-nonspreading linear three-dimensional wave packets. *Opt. Lett.* **36**, 2176-2178 (2011).
2. Lotti, A., Couairon, A., Faccio, D. & Trapani, P. D. Energy-flux characterization of conical and space-time coupled wave packets. *Phys. Rev. A* **81**, 023810 (2010).
3. Wan, C., Cao, Q., Chen, J., Chong, A. & Zhan, Q. Toroidal vortices of light. *Nat. Photonics***16**, 519-522 (2022).
4. Levy, U., Derevyanko, S. & Silberberg, Y. Light modes of free space. *Progress in Optics*. **61**, 237-281 (2016).
5. Goodman, J.W. *Introduction to Fourier Optics* (Roberts and Co, 2005).
6. Agrawal, G. P. *Nonlinear Fiber Optics* (Academic Press, 2012).
7. Gradshteyn, I. S. & Ryzhik, I. M. *Table of integrals, series, and products* (Academic press, 2014).
8. Allen, L., Beijersbergen, M. W., Spreeuw, R. J. C. & Woerdman, J. P. Orbital angular momentum of light and the transformation of Laguerre-Gaussian laser modes. *Phys. Rev. A* **45**, 8185-8189 (1992).
9. Bliokh, K.Y. & Nori, F. Spatiotemporal vortex beams and angular momentum. *Phys. Rev. A* **86**, 033824 (2012).
10. Chong, A., Wan, C., Chen, J. & Zhan, Q. Generation of spatiotemporal optical vortices with controllable transverse orbital angular momentum. *Nat. Photon.* **14**, 350-354 (2020).
11. Schmidt, J. D. *Numerical Simulation* Torner, L., Torres, J.P. and Carrasco, S.*of Optical Wave Propagation with Examples in MATLAB* (SPIE, 2010).
12. Liu, X., Li, Z., Monfared, Y. E., Liang, C., Wang, F., Hoenders, B. J., Cai, Y. & Ma, P. Flexible autofocusing properties of ring Pearcey beams by means of a cross phase. *Opt. Lett.* **46**, 70-73 (2021).
13. Abramochkin, E. & Volostnikov, V. Beam transformations and nontransformed beams. *Opt. Commun.* **83**, 123–135 (1991).
14. Li, H., Bazarov, I. V., Dunham, B. M. & Wise, F. W. Three-dimensional laser pulse intensity diagnostic for photoinjectors. *Phys. Rev. ST Accel. Beams* **14**, 112802 (2011).
15. Torner, L., Torres, J. P. & Carrasco, S. Digital spiral imaging. *Opt. Express* **13**, 873-881 (2005).
16. Yang, Y., Zhao, Q., Liu, L., Liu, Y., Rosales-Guzmán, C. & Qiu, C. W. Manipulation of orbital-angular-momentum spectrum using pinhole plates. *Phys. Rev. Appl.* **12**, 064007 (2019).
