# Supplementary material for: Spatiotemporal optical vortices with controllable radial and azimuthal quantum numbers
Source: Nat Commun. 2024 Jun 27;15:5435. doi: 10.1038/s41467-024-49819-4 (PMC11211508; doi:10.1038/s41467-024-49819-4)
Supplement: Supplementary file 3 — Description of Additional Supplementary Files [file 41467_2024_49819_MOESM3_ESM.docx]

**Description of Additional Supplementary Files**

**Supplementary Movie 1:** Experimentally representative 3D reconstruction process for STLG wavepacket with p=2 and l=+1, in which the time step is set to be ~33fs.

**Supplementary Movie 2:** Numerical simulation for the topological evolution of space-time mode conversion between STLG (p=1 and l=+2) wavepacket and STHG wavepacket under different spatiotemporal astigmatism
